# Supplementary material for: Accessing acute care hospitals in the San Francisco Bay Area after a major hayward earthquake
Source: Nat Commun. 2025 Oct 22;16:9328. doi: 10.1038/s41467-025-64354-6 (PMC12546628; doi:10.1038/s41467-025-64354-6)
Supplement: Supplementary file 1 — Supplementary Information [file 41467_2025_64354_MOESM1_ESM.pdf]

Accessing Acute Care Hospitals in the San Francisco Bay after a Major Hayward Earthquake

Supplementary Information

Ceferino et al.

Supplementary Note 1

We processed the inventory data for 76 acute care hospitals in the Bay Area (Figure 1). Acute care hospitals provide inpatient medical care and other related services for surgery, acute medical conditions, or injuries (usually for a short-term illness or condition). Thus, these hospitals are critical for emergencies, e.g., such as those after earthquakes. The data contains information on the hospitals' 426 buildings, including their locations, structural typologies, year of construction, number of stories, and vulnerability ratings.[1]

We also found that old and vulnerable buildings are still functional. 19% of buildings were constructed before 1973 when California passed the Alfred E. Alquist Hospital Facilities Seismic Safety Act requiring more stringent design for hospitals.[2] Moreover, 59% of buildings were built before 1994, when the Northridge Earthquake revealed that welded and bolted connections were non-ductile in steel buildings. In 1994, the Alquist Act was amended to establish structural and non-structural vulnerability categories.

The building portfolio has various structural systems. A few buildings report more than one structural system as they can be mixed or be different for orthogonal directions. This paper focused only on the primary structural type, which is reported for all buildings (Supplementary Figure 1). Most hospital buildings are steel moment frames (S1), followed by concrete shear walls (C2), and steel braced frames (S2), with 38%, 21%, and 17%, respectively. Noticeably, California is building fewer new C2 hospitals. Before 1974 (pre-Alquist Act), there were 56 buildings, but between 1974 and 1994 (pre-Northridge), there were 23 new hospitals, and after 1994 only 11 new ones. Conversely, there are more newer steel hospitals (S1 and S2). Before 1974, there were only seven buildings, but between 1974 and 1994, there were 112 new hospital buildings, and after 1994 119 new ones.

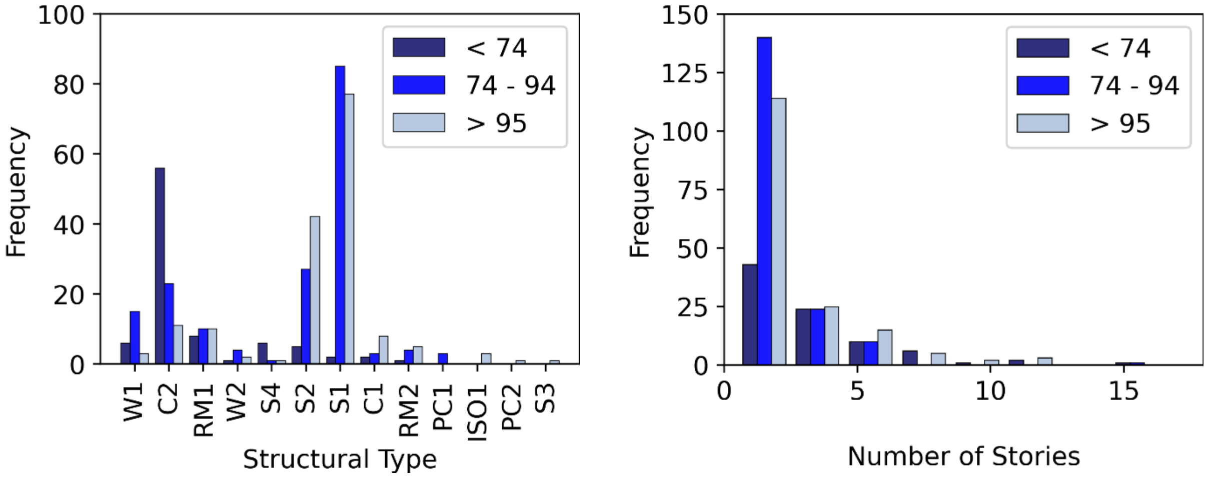

Supplementary Figure 1. *Hospital Exposure in the San Francisco Bay Area. Left: Histogram of buildings' structural types. Right: Histogram of the buildings' number of stories. W1: Wood, Light Frame, W2: Commercial and Industrial, C1: Concrete Moment Frame, C2: Concrete Shear Walls, S1: Steel Moment Frame, S2: Steel Braced Frame, S3: Steel Light Frame, S4: Steel Frame with Cast-in-Place Concrete Shear Walls, RM1: Reinforced Masonry Bearing Walls with Wood or Metal Deck Diaphragm, RM2: Reinforced Masonry Bearing Walls with Precast Concrete Diaphragms, PC1: Precast Concrete Tilt-Up Walls, PC2: Pre-cast Concrete Frames with Concrete Shear Walls, ISO1: Triple Concave Friction Pendulum Isolators. Year of construction is indicated in different colors.*

The average number of stories is 2.4, with a standard deviation of 2.2% (Supplementary Figure 1). Most buildings have 1- and 2- stories, 53% and 17%, respectively, highlighting a prevalence of structures with short periods of vibration (low-story buildings). Only eight buildings have more than 10 stories, and the tallest building has 15 stories. The only way to transport patients undergoing surgery in a hospital is by using the elevator, which can become a bottleneck. Usually, hospitals are laid out to minimize transporting surgical patients on the elevators.

Supplementary Note 2

The ground shaking from earthquakes in recent earthquakes has been significantly lower than that from the M 7.9 San Francisco Earthquake in 1906.

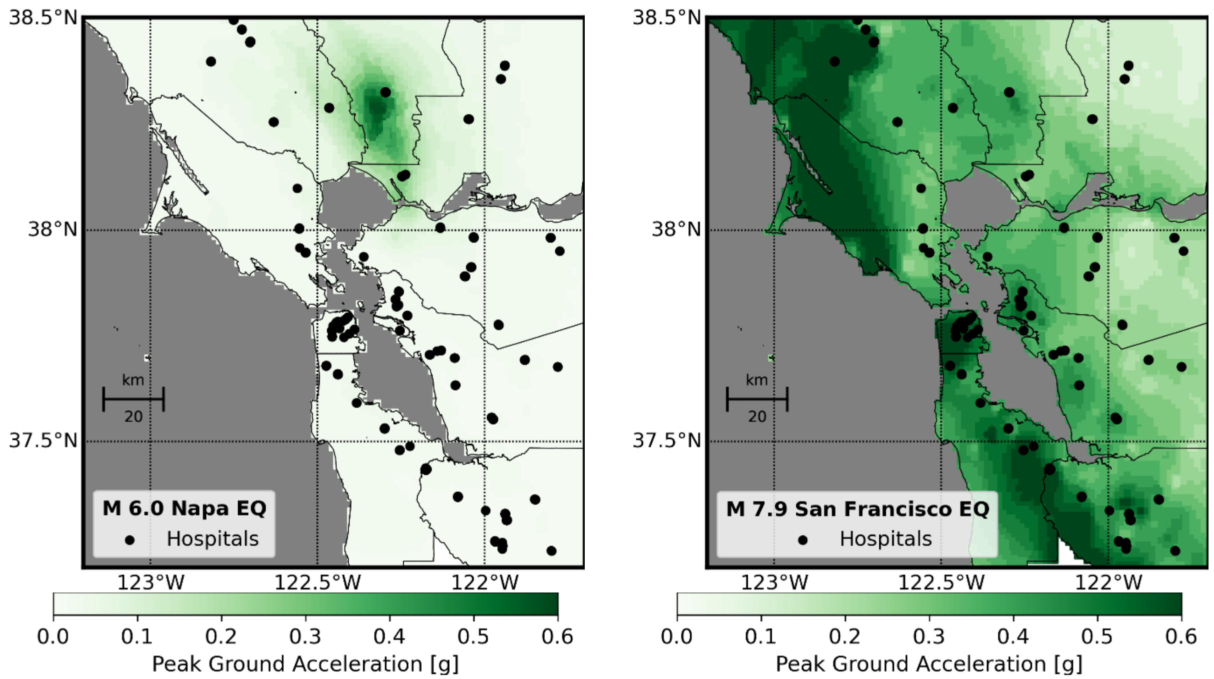

Supplementary Figure 2. *Peak ground accelerations for two historical earthquakes in the Bay Area, demonstrating that most of the acute care portfolio (and the Bay Area communities) have not experienced large shaking intensities in more than a century. Left: The 2016 M 6.0 Napa Earthquake. Map data from [OpenStreetMap © contributors](). Right: The great 1906 M 7.9 San Francisco Earthquake. Map data from OpenStreetMap.*

Supplementary Note 3

Many hospitals are close to the Hayward Fault and have high infrastructure vulnerabilities (Supplementary Figure 3). From the 214 buildings with probability above 0.25 of structural damage, 151 (71%) buildings are either 20 km or less from the rupture or have an SPC of 1 or 2. Of the 254 buildings with probability above 0.25 of non-structural damage, 238 (94%) buildings are either 20 km or less from the rupture or have an NPC of 1, 2, or 3.

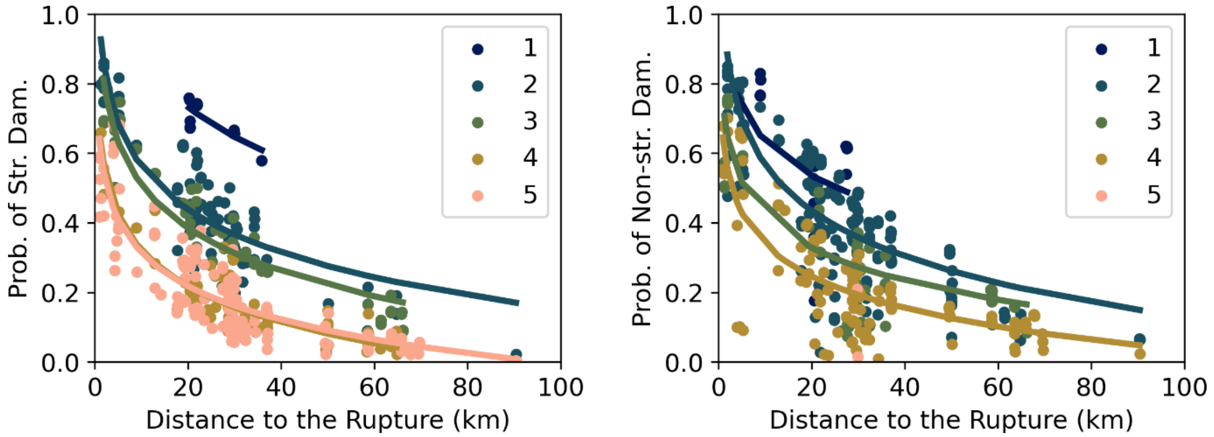

Supplementary Figure 3. *Earthquake damage simulations for hospital buildings. Left: Estimated damage probabilities on buildings' structural components for different SPCs (1 to 5). Right: Estimated probabilities of damage on buildings' non-structural components for different NPCs (1 to 5). We used 5,000 Monte Carlo simulations to conduct the analysis.*

Supplementary Note 4

We also studied the sensitivity of hospital functionality to different damage thresholds for each county in the Bay Area to complement Table 1 and Figure 3 in the article.

Supplementary Table 1. *Predicted post-earthquake hospital bed capacities across Bay Area counties under thresholds of moderate (favorable) and extensive (idealistic) damage scenarios. The numbers in parentheses indicate the percentage in proportion to the pre-earthquake capacity.*

	Favorable		Idealistic	
**County**	**Mean**	**Std. Dev.**	**Mean**	**Std. Dev.**
Alameda	1,743 (51%)	748 (24%)	2,635 (82%)	554 (17%)
Contra Costa	1,372 (75%)	378 (22%)	1,649 (94%)	188 (11%)
Marin	482 (71%)	159 (25%)	581 (93%)	84 (13%)
Napa	319 (89%)	50 (14%)	345 (98%)	19 (5%)
San Francisco	3,258 (89%)	502 (14%)	3,547 (98%)	173 (5%)
San Mateo	1,269 (86%)	229 (16%)	1,390 (96%)	120 (8%)
Santa Clara	3,512 (80%)	725 (17%)	4,068 (94%)	346 (8%)
Solano	636 (88%)	116 (16%)	705 (98%)	50 (7%)
Sonoma	537 (90%)	64 (11%)	573 (98%)	26 (4%)
**Total**	**13,129 (79%)**	**2,583 (16%)**	**15,492 (93%)**	**1,271 (8%)**

Supplementary Figure 4 shows the functionality ratio, i.e., the ratio between the expected number of functional beds after the earthquake and the pre-earthquake conditions (see Methods), as a function of the distance to the rupture. Hospital functionality is significantly lower within 20 km from the rupture, making counties like Alameda particularly at high risk. The average functionality ratio is 25%, 51%, and 82% for hospitals within 20km, between 20 and 40 km, and beyond 40 km, respectively.

Supplementary Figure 4 highlights the hospitals with a high share of buildings with poor SPCs or NPCs to underline that those physical vulnerabilities cascade to significant reductions in hospital capacity. Hospitals with more than 50% of their buildings with poor SPC (i.e., SPC ≤ 2) have a 14% smaller functionality ratio than the others, i.e., 40% versus 54%. Hospitals with more than 50% of their buildings with poor NPC (i.e., NPC ≤ 3) among their buildings have a 17% smaller functionality ratio than the others, i.e., 45% versus 62%.

We analyzed all simulations that led to hospital functionality ratios below 50% to evaluate the percentage of buildings with structural and non-structural damage (see Methods). Supplementary Figure 4 shows that the contributions of structural and non-structural damage to hospital disruptions vary per the distance to the rupture. Within the first 20 km, hospitals with large disruptions have, on average, 64% and 70% of their buildings with structural and non-structural damage, respectively. At larger distances, however, the relative contributions of non-structural damage increase. For example, beyond 40 km, hospitals with large disruptions have, on average, 30% and 38% of their buildings with structural and non-structural damage, respectively. While the relative contributions from the structural components were slightly smaller than non-structural components, with a ratio of 0.91 (64% versus 70%) within the first 20 km, the ratio of 0.77 (30% versus 38%) beyond 40 km indicates that non-structural component strength is important for hospital functionality even

at long distances from the rupture. Earthquakes can damage non-structural components over large geographical extents because the buildings' dynamics can amplify peak floor accelerations, even at long distances.

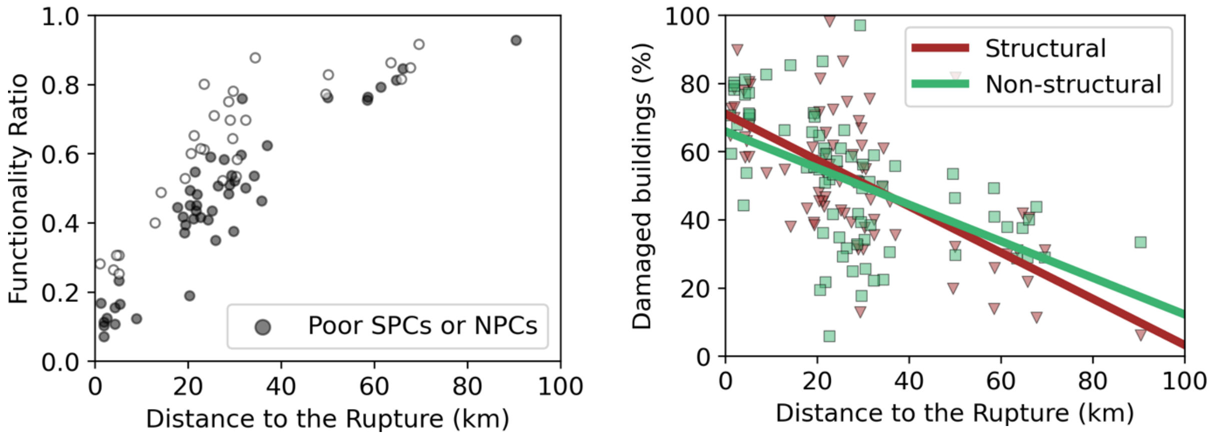

Supplementary Figure 4. *Hospital risk versus distance to the earthquake rupture. Left: Expected functionality ratio for each hospital for the slight damage threshold, indicating those hospitals with the top share (top quartile) of low SPCs (i.e., one and two) and NPCs (i.e., one, two, and three). Right: (De-aggregated) percentages of buildings with structural and non-structural damage given that the hospital has high functionality loss (i.e., more than 50%).*

.

Supplementary Note 5

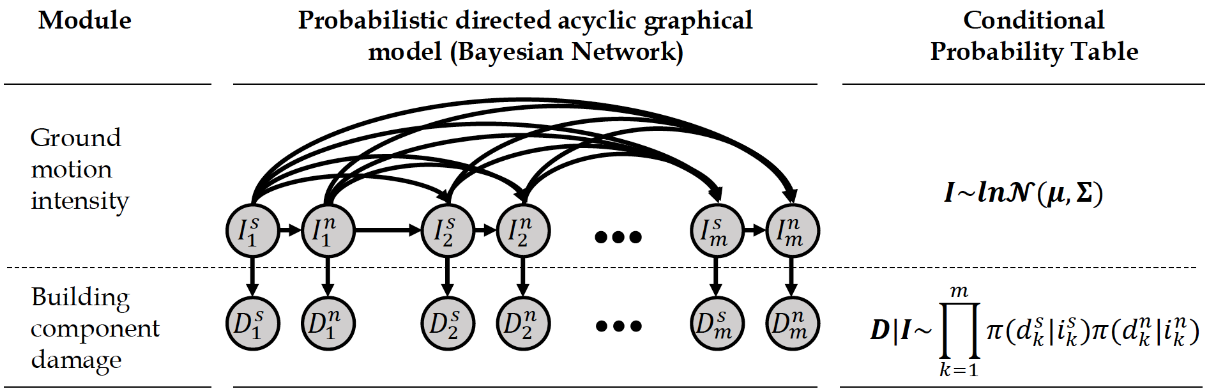

I_k^s: Ground motion intensity for structural damage at the location of the k-th building.
I_k^n: Ground motion intensity for non-structural damage at the location of the k-th building.
D_k^s: Structural damage on the k-th building.
D_k^n: Non-structural damage on the k-th building.
Arrows: Probabilistic dependencies (directed edges in the Bayesian Network).
Gray circles: Random variables representing uncertain quantities.

Supplementary Figure 5. *Bayesian Network Representation of Earthquake-Induced Building Damage. The Probabilistic graphical model (Bayesian network) represents the joint distribution of the risk model to assess structural and non-structural damage from earthquake ground motion intensities.*

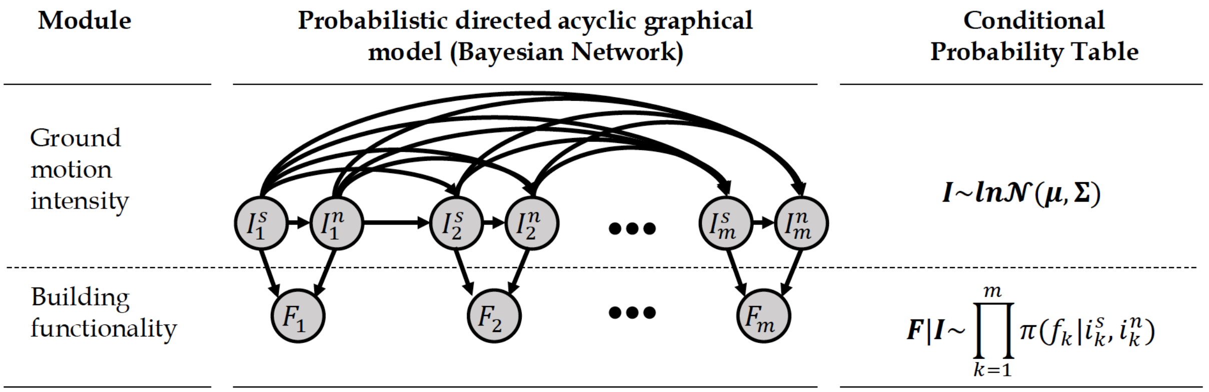

I_k^s: Ground motion intensity for structural damage at the location of the k-th building.
I_k^n: Ground motion intensity for non-structural damage at the location of the k-th building.
F_k: Functionality of the k-th building.
Arrows: Probabilistic dependencies (directed edges in the Bayesian Network).
Gray circles: Random variables representing uncertain quantities.

Supplementary Figure 6. *Bayesian Network Representation of Earthquake-Induced Building Functionality. The probabilistic graphical model (Bayesian network) represents the joint distribution of the risk model to assess hospital functionality from earthquake ground motion intensities.*

Supplementary References

1. California Health and Human Services. Hospital Building Data. https://data.chhs.ca.gov/dataset/hospital-building-data (2023).
2. Preston, B. *et al. Updating the Costs of Compliance for California's Hospital Seismic Safety Standards*. *Updating the Costs of Compliance for California's Hospital Seismic Safety Standards* (RAND Corporation, 2019). doi:10.7249/rr3059.
